# Pyogenic Spondylitis Caused by Parvimonas micra: A Case Report

**DOI:** 10.7759/cureus.48665

**Published:** 2023-11-11

**Authors:** Saneyuki Itagaki, Tsuneaki Kenzaka

**Affiliations:** 1 Department of General Medicine, Toyooka Public Hospital, Toyooka, JPN; 2 Department of Internal Medicine, Hyogo Prefectural Tamba Medical Center, Tanba, JPN; 3 Division of Community Medicine and Career Development, Kobe University Graduate School of Medicine, Kobe, JPN

**Keywords:** case report, back pain, gram-positive anaerobic coccus, pyogenic spondylitis, parvimonas micra

## Abstract

*Parvimonas micra* (*P. micra*) is a gram-positive anaerobic coccus endemic to the oral cavity and intestinal tract. We report a case of pyogenic spondylitis caused by *P. micra* and summarize the clinical features of previous case reports. An 81-year-old man with a history of lumbar vertebral compression fracture two years previously presented to the emergency department with low back pain. He was clinically diagnosed with pyogenic spondylitis due to difficulty in moving his body, spinal tapping pain, and signs of inflammation. He was hospitalized, and aerobic and anaerobic blood culture samples were collected, but the results were negative. Computed tomography and magnetic resonance imaging revealed inflammation in the second and third lumbar vertebrae and L2/3 and L3/4 intervertebral discs, and culture of the infected disc biopsy showed *P. micra* growth. After six weeks of treatment with ampicillin-sulbactam and ampicillin, the patient's symptoms improved, and he was discharged. During hospitalization, he was diagnosed with periodontitis and type 2 diabetes; his dentures were adjusted, and he was started on an oral hypoglycemic agent. Pyogenic spondylitis caused by *P. micra* tends to be associated with oral infections. This case illustrates the importance of appropriate detection and treatment of the source of infection to prevent recurrence.

## Introduction

*Parvimonas micra* (*P. micra*) is a gram-positive anaerobic coccus that is part of the oral and intestinal flora [1]. It can cause periodontitis and various infections [2], including spondylitis, skin infections, peritonsillar abscesses, and pulmonary pyogenic disease [3]. Typically, it is found in mixed polymicrobial infections rather than as a single isolate. Risk factors for* P. micra* infection include oral procedures, such as denture fitting and dental treatment, and systemic diseases, such as diabetes mellitus. The incidence of pyogenic spondylitis caused by *P. micra *is low [3]. We report a case of pyogenic spondylitis caused by *P. micra* and review previous case reports of pyogenic spondylitis caused by *P. micra*.

## Case presentation

An 81-year-old man presented with a two-month history of increasing back pain. He did not seek medical care until one day before admission, when he became immobile and was taken to the emergency department. He had a history of a lumbar vertebral compression fracture two years previously. Further, his medical history included hypertension, benign prostatic hyperplasia, and Lewy body disease. He reported fever and back pain but did not report any respiratory or gastrointestinal symptoms, headache, abdominal pain, or urinary or fecal incontinence.

Investigations

The patient was alert and orientated, with the following findings: blood pressure 131/71 mmHg, pulse 70 beats/min (regular), temperature 36.9°C, respiratory rate 12 breaths/min, and oxygen saturation 98% (breathing room air). No hemorrhagic spots were observed on the conjunctiva, and chest auscultation revealed normal heart and respiratory sounds. The abdomen was flat with no tenderness, but tapping pain was present in the lumbar spine. No oral or dental lesions were observed.

Hematology revealed a leukocyte count of 23700 cells/μL with 94.4% neutrophils, 4.0% lymphocytes, and 1.7% monocytes. Blood biochemistry revealed aspartate aminotransferase level 133 U/L, alanine aminotransferase level 35 U/L, creatine phosphokinase level 6314 U/L, blood urea nitrogen level 22.4 mg/dL, creatinine level 0.80 mg/dL, C-reactive protein level 7.3 mg/dL, and glycated hemoglobin level 6.8%.

**Figure 1:**
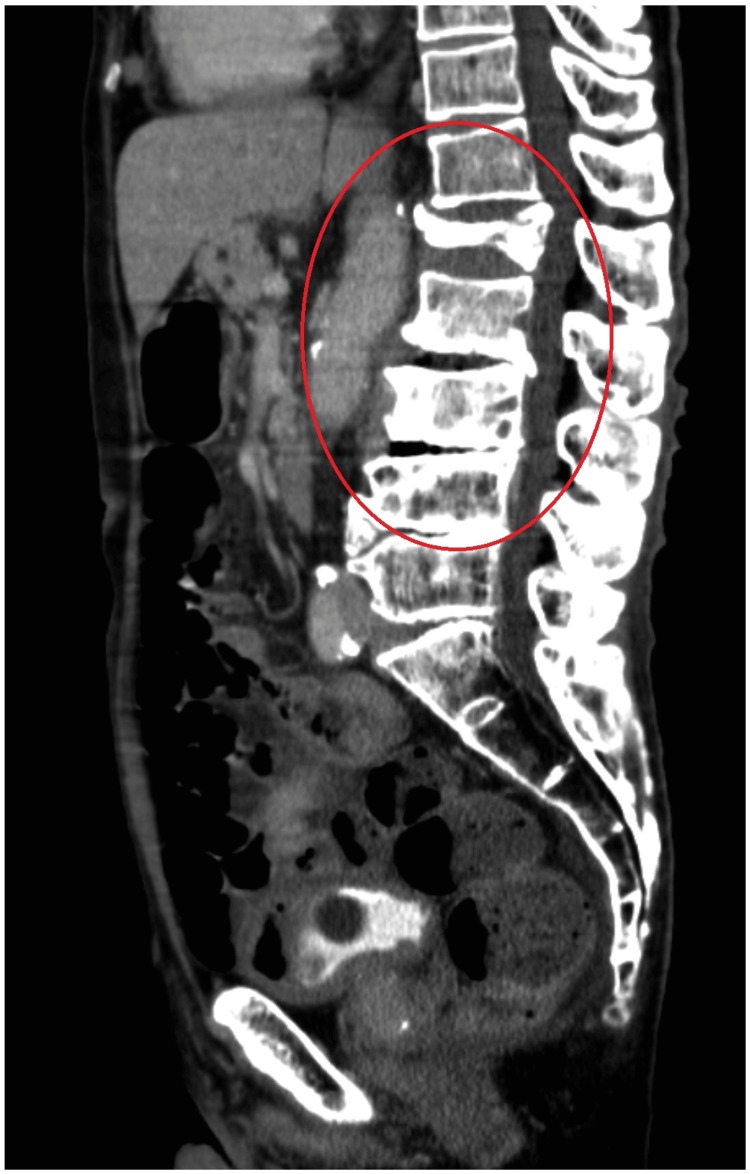
Computed tomography image of the lumbar spine Computed tomography image of the lumbar spine showing gas production in the L2/3 and L3/4 discs (red circle)

**Figure 2:**
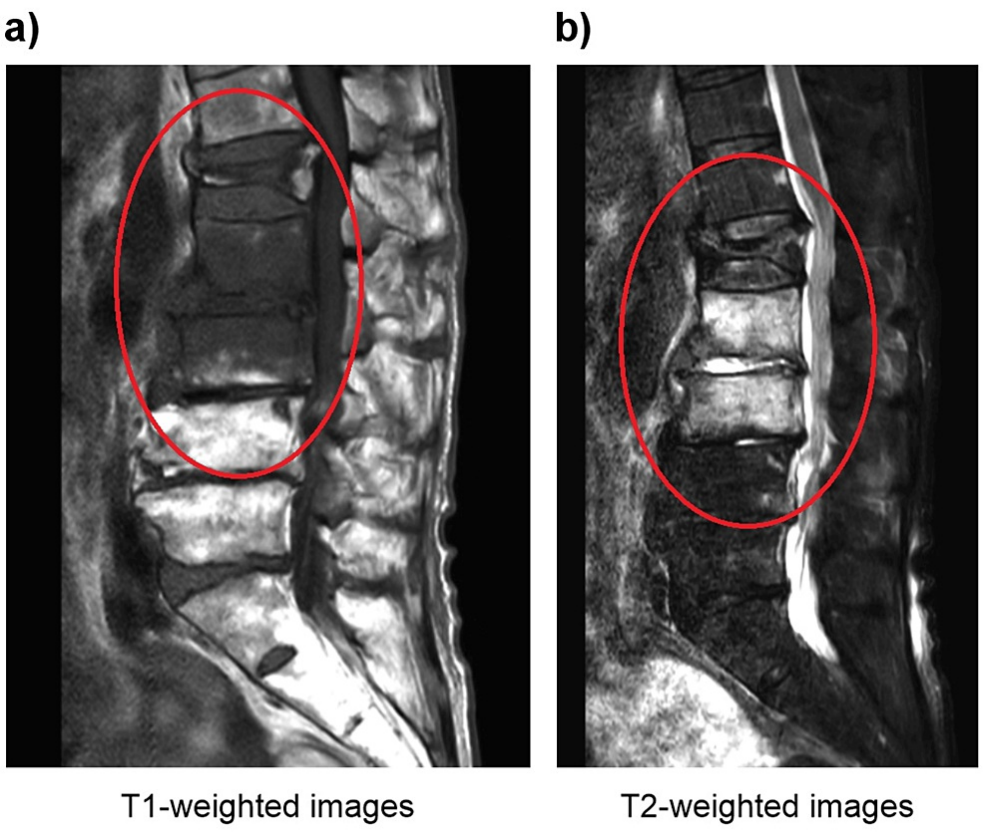
Magnetic resonance imaging without contrast of the lumbar spine Magnetic resonance imaging without contrast of the lumbar spine showing fluid accumulation in the L2/3 and L3/4 discs and (a) low-signal changes in the L2 and L3 vertebrae on T1-weighted imaging and (b) high-signal changes in the L2 and L3 vertebrae on T2-weighted imaging. An old compression fracture can be seen in L1 (red circles)

Contrast-enhanced computed tomography of the thoracoabdominal region revealed gas production in the L2/3 and L3/4 intervertebral discs and contrast effects in the surrounding iliopsoas muscle (Figure 1). Simple lumbar spine magnetic resonance imaging (MRI) showed fluid retention in the L2/3 and L3/4 discs. The L2 and L3 vertebrae showed low-signal changes on T1-weighted imaging and high-signal changes on T2-weighted imaging (Figure 2).

Differential diagnosis

The conditions considered in the differential diagnosis included pyogenic spondylitis, bacteremia including infective endocarditis, non-pyogenic spondylitis associated with collagen disease and other conditions, as well as multiple myeloma.

Diagnosis and treatment

Based on the patient's symptoms of fever, back pain, elevated inflammatory marker levels, and signs of inflammation observed on lumbar spine imaging, he was clinically diagnosed with pyogenic spondylitis. Considering the possibility of a blood-borne infection, blood culture samples were collected, and the patient was hospitalized for observation. However, both the aerobic and anaerobic blood culture results were negative. On day six of hospitalization, an L2/3-disc puncture was performed, and purulent fluid was collected. A culture of the puncture fluid from the spinal abscess revealed growth of gram-positive anaerobic cocci, confirming the diagnosis of pyogenic spondylitis. Intravenous administration of ampicillin-sulbactam (3 g every six hours) was initiated. 

**Figure 3:**
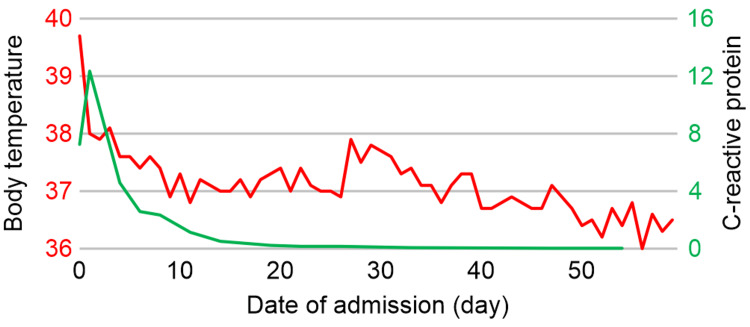
Course after hospitalization Course after hospitalization showing changes in body temperature (left axis, red line) and C-reactive protein level (right axis, green line)

The fluid sample was inoculated in aerobic and anaerobic blood agar (BD Columbia Agar 5% Sheepblood®; Becton Dickinson, Franklin Lakes, New Jersey), chocolate agar (BD Choco Agar; Becton Dickinson), and thioglycollate broth (BDTM Fluid Thioglycollate Medium; Becton Dickinson); all these cultures were incubated at 37ºC. On day three of incubation, the growth of gram-positive cocci in small chains was observed only in the anaerobic blood agar. White and smooth colonies were observed and identified as *P. micra* using matrix-assisted laser desorption ionization-time of flight mass spectrometry (MALDI-TOF MS), with an identification score of >2.0. Further, the 16S rRNA sequence of the isolate was determined, and a homology search was performed, revealing a 99.4% identity (1460 bp out of 1469 bp) with the known sequence of *P. micra*. Notably, multiple myeloma was ruled out based on further pathology and diagnostic assessments. Regarding antimicrobial susceptibility, the minimum inhibitory concentrations (mg/mL) were as follows: penicillin-G ≤0.5, ampicillin ≤0.5, piperacillin ≤8, ampicillin-sulbactam ≤2, cefotiam ≤4, cefmetazole ≤4, clindamycin ≤0.5, minocycline ≤1, imipenem ≤1, and levofloxacin ≤1. Consequently, the patient's treatment was continued with ampicillin monotherapy (2 g every six hours) based on antimicrobial susceptibility testing results. The patient's fever, inflammatory marker levels (Figure 3), and back pain resolved, and he was discharged after six weeks of intravenous antibiotic therapy. Throughout the hospitalization period, the patient remained asymptomatic, but the presence of dentures and denture maladjustment was considered to be the entry portal for *P. micra*. Therefore, tooth extraction and denture repair were performed. The patient was also diagnosed with type 2 diabetes mellitus and was started on an oral hypoglycemic agent.

Outcome and follow-up

**Figure 4:**
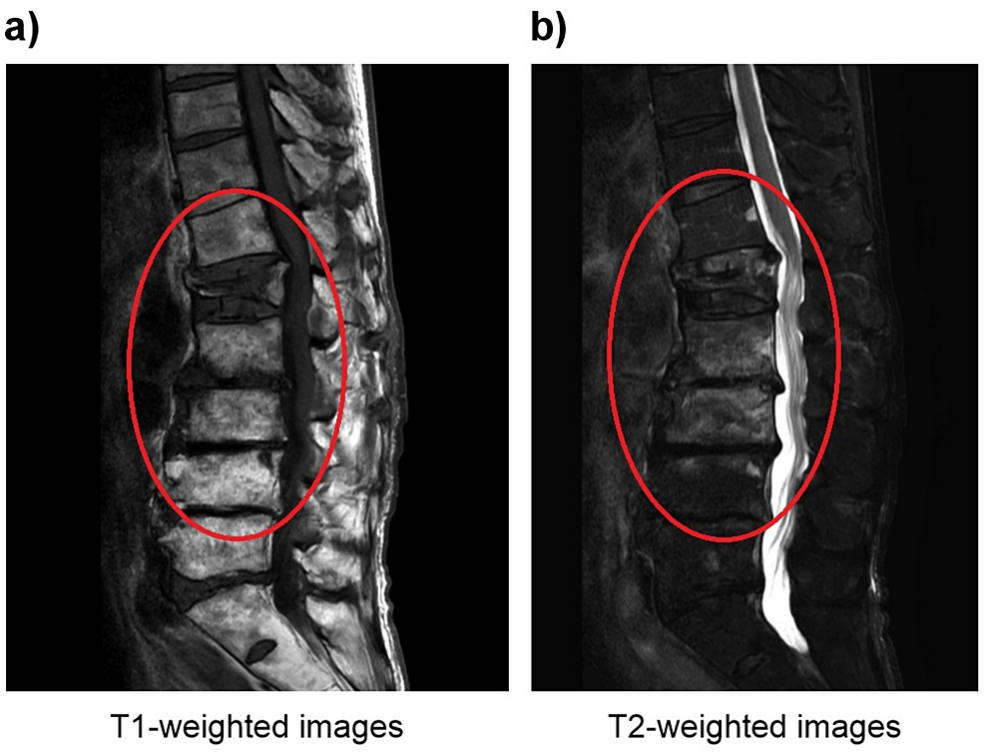
Magnetic resonance imaging without contrast of the lumbar spine Magnetic resonance imaging without contrast of the lumbar spine was performed three months after discharge, showing the resolution of the fluid accumulation and signal changes. The L2 and L3 vertebrae showed recovery of signal change on (a) T1-weighted images and (b) on T2-weighted images. An old compression fracture can be seen in L1 (red circle).

At a follow-up visit one month after discharge, the patient had no fever or recurrence of back pain. MRI performed three months after discharge showed that the signal changes in the vertebral body were attenuated (Figure 4), and the pyogenic spondylitis was considered cured.

## Discussion

Gram-positive cocci are the most common causative organisms of pyogenic spondylitis, with *Staphylococcus aureus* accounting for 30-80% of the cases. Other causative organisms include *Escherichia coli* and *Mycobacterium tuberculosis* [4]. Pyogenic spondylitis caused by *P. micra* is rare.

We conducted a MEDLINE search for published reports on vertebral osteomyelitis caused by *P. micra *using the search terms "*Parvimonas micra*" and "vertebral osteomyelitis" on November 6, 2023. The literature search yielded 16 references reporting 25 cases [5-20] (Table 1).

**Table 1:** Summary of published case reports of vertebral osteomyelitis caused by Parvimonas micra ABPC/SBT - ampicillin/sulbactam; AMK - amikacin; AMPC - ampicillin; CFPX - ciprofloxacin; CLDM - clindamycin; CTRX - ceftriaxone; GM - gentamicin; GNR - gram-negative rods; MNZ - metronidazole; PCG - benzylpenicillin; PIPC/TAZ - piperacillin/tazobactam; RFP - rifampicin; TEIC - teicoplanin; VCM - vancomycin

Case	Author, reference	Age (years), sex	Infected tissue	Source of infection	Mixed infection	Diabetes mellitus	Therapy	Outcome
1	Duijvenbode et al. [5]	78, M	Bone	Tooth extraction	No	No	Surgical decompression, VCM, CFPX, PCG, CLDM	Full recovery after 6 months
2	Papasian et al. [6]	70, M	Biopsy	Unknown	No	No	Surgical debridement, CLDM	Full recovery after 5 months
3	Leder et al. [7]	70, M	Cerebrospinal fluid	Ulcerative colitis	No	No	PCG, AMPC, MNZ	Full recovery after 1 year
4	García González et al. [8]	62, M	Unknown	Unknown	No	Yes	CLDM	Full recovery
5	Uemura et al. [9]	84, M	Bone	Periodontitis	Yes, GNR	No	ABPC/SBT	Full recovery
6	Uemura et al. [9]	85, F	Blood	Periodontitis	Yes, Fusobacterium nucleatum	No	ABPC/SBT	Unknown
7	Dahya et al. [10]	62, M	Aortic valve	Infectious endocarditis	No	No	VCM, CTRX	Full recovery after 6 months
8	Pilmis et al. [11]	83, M	Blood	Unknown	No	No	AMPC, GM	Full recovery after 6 months
9	Medina et al. [12]	23, F	Cerebrospinal fluid	Unknown	No	No	AMPC/CVA, RFP, CLDM	Full recovery after 3 months
10	Endo et al. [13]	55, F	Bone	Dental treatment	No	No	Surgical debridement, ABPC/SBT, MNZ	Full recovery
11	Gahier et al. [14]	59, F	Blood	Periodontitis	No	No	GM, MNZ, AMPC	Full recovery
12	Gahier et al. [14]	82, F	Blood	Periodontitis	No	No	CTRX, GM, AMPC	Full recovery
13	Gahier et al. [14]	60, F	Blood	Unknown	No	No	CTRX, GM, AMPC	Full recovery
14	George et al. [15]	49, M	Bone	Dental treatment	Yes	No	Surgical treatment, CTRX	Full recovery after 3 months
15	Jones et al. [16]	72, M	Intervertebral disc	Tooth extraction	Yes, Propionibacterium acnes	No	PIPC/TAZ, AMPC/CVA	Full recovery after 1 year
16	Jones et al. [16]	72, F	Pus from an abscess	Unknown	Yes	No	PIPC/TAZ	Full recovery after 5 months
17	Higashi et al. [17]	67, M	Intervertebral disc	Periodontitis	No	Yes	ABPC/SBT	Full recovery after 3 months
18	Cleaver et al. [18]	45, F	Intervertebral disc	Unknown	Yes, Fusobacterium sp.	No	TEIC, PIPC/TAZ, AMK	Full recovery after 6 weeks
19	Yoo et al. [19]	77, F	Blood, intervertebral disc	Alveolar pyorrhea	No	No	CTRX, MNZ	Full recovery after 3 months
20	Durovic et al. [20]	82, M	Blood	Unknown	No	No	Surgical treatment, AMPC/CVA	Death
21	Durovic et al. [20]	69, M	Intervertebral disc	Tooth extraction	No	Yes	AMPC/CVA	Full recovery after 6 months
22	Durovic et al. [20]	72, M	Blood, abscess	Dental treatment	No	Yes	Surgical debridement, AMPC/CVA, PCG	Full recovery after 2 months
23	Durovic et al. [20]	72, F	Blood	Unknown	Yes, Fusobacterium nucleatum	No	AMPC/CVA, MFLX	Unknown
24	Durovic et al. [20]	72, M	Blood	Unknown	No	Yes	PCG, CLDM	Full recovery after 6 months
25	Durovic et al. [20]	63, M	Blood	Periodontitis	No	No	AMPC/CVA, AMPC	Full recovery after 5 months
26	This case	81, M	Intervertebral disc	Tooth decay	No	Yes	ABPC/SBT, ABPC	Full recovery after 3 months

The patients included 15 men and 11 women with a mean age of 67.9 ± 13.7 years. Oral infections and dental procedures were cited as the route of infection in 14 cases (54%), whereas no association was noted with dental infections or treatment in the other 10 cases. Overall, seven cases (27%) were polymicrobial infections with other organisms, and surgical treatment was required in six cases (23%). Four patients (15%) had underlying diabetes mellitus, and one patient (4%) died due to a ruptured aortic aneurysm.

The incidence of pyogenic spondylitis increased in Japan from 5.3 per 100,000 population in 2007 to 7.4 per 100,000 population in 2010. This condition is a rare but life-threatening disease with an in-hospital mortality rate of 6.0% [21].

Patients suspected of having pyogenic spondylitis should undergo a blood culture, which has a positivity rate of approximately 50%. Image-guided aspiration biopsy is recommended if the causative organism cannot be identified by blood culture [22], as in this case.

In our case, identification of the causative organism facilitated the investigation of comorbidities such as periodontitis and type 2 diabetes mellitus based on characteristics of the causative bacteria.

## Conclusions

This case report presents a case of pyogenic spondylitis due to a rare causative agent, *P. micra*. We reviewed previous cases of pyogenic spondylitis caused by *P. micra*. As pyogenic spondylitis caused by *P. micra* often occurs as a complication of oral infections, oral examination is essential in the diagnostic process.

## References

[1] Rams TE, Feik D, Listgarten MA, Slots J (1992). Peptostreptococcus micros in human periodontitis. Oral Microbiol Immunol.

[2] Didilescu AC, Rusu D, Anghel A (2012). Investigation of six selected bacterial species in endo-periodontal lesions. Int Endod J.

[3] Cobo F, Rodríguez-Granger J, Sampedro A, Aliaga-Martínez L, Navarro-Marí JM (2017). Pleural effusion due to Parvimonas micra. A case report and a literature review of 30 cases. Rev Esp Quimioter.

[4] Duarte RM, Vaccaro AR (2013). Spinal infection: state of the art and management algorithm. Eur Spine J.

[5] van Duijvenbode DC, Kuiper JW, Holewijn RM, Stadhouder A (2018). Parvimonas micra spondylodiscitis: A case report and systematic review of the literature. J Orthop Case Rep.

[6] Papasian CJ, McGregor DH, Hodges GR, Kennedy J (1986). Peptostreptococcal vertebral osteomyelitis. J Clin Microbiol.

[7] Leder KS, Barlam TF (2000). A case of paraspinal abscess and diskitis due to Peptostreptococcus micros. Clin Infect Dis.

[8] García González M, Muñiz Montes JR, García Rosado D, Bustabad Reyes S (2014). Multifocal hematogenous vertebral osteomyelitis due to Parvimonas micra and a subsequent pleural effusion in a diabetic patient. Reumatol Clin.

[9] Uemura H, Hayakawa K, Shimada K (2014). Parvimonas micra as a causative organism of spondylodiscitis: a report of two cases and a literature review. Int J Infect Dis.

[10] Dahya V, Chalasani P, Ramgopal M (2015). Peptostreptococcus endocarditis presenting as lumbar discitis in an immunocompromised patient. Am J Med Sci.

[11] Pilmis B, Israel J, Le Monnier A, Mizrahi A (2015). Spondylodiscitis due to anaerobic bacteria about a case of Parvimonas micra infection. Anaerobe.

[12] Medina F, Tatay M, Smati M, Aoun O, Tankovic J, Bouchaud O, Méchaï F (2015). Lemierre's syndrome: an unusual presentation. Med Mal Infect.

[13] Endo S, Nemoto T, Yano H (2015). First confirmed case of spondylodiscitis with epidural abscess caused by Parvimonas micra. J Infect Chemother.

[14] Gahier M, Cozic C, Bourdon S, Guimard T, Cormier G (2015). Spinal infections caused by Parvimonas micra. Med Mal Infect.

[15] George IA, Pande A, Parsaei S (2015). Delayed infection with Parvimonas micra following spinal instrumentation. Anaerobe.

[16] Jones SL, Riordan JW, Glasgow AL, Botes J, Boutlis CS (2015). Two cases of spondylodiscitis caused by Parvimonas micra. Intern Med J.

[17] Higashi Y, Nakamura S, Niimi H (2017). Spondylodiscitis due to Parvimonas micra diagnosed by the melting temperature mapping method: a case report. BMC Infect Dis.

[18] Cleaver LM, Palanivel S, Mack D, Warren S (2017). A case of polymicrobial anaerobic spondylodiscitis due to Parvimonas micra and Fusobacterium nucleatum. JMM Case Rep.

[19] Yoo LJ, Zulkifli MD, O'Connor M, Waldron R (2019). Parvimonas micra spondylodiscitis with psoas abscess. BMJ Case Rep.

[20] Durovic A, Eberhard N, Schären S, Widmer AF (2020). Parvimonas micra as a rare cause of spondylodiscitis - case series from a single centre. Swiss Med Wkly.

[21] Akiyama T, Chikuda H, Yasunaga H, Horiguchi H, Fushimi K, Saita K (2013). Incidence and risk factors for mortality of vertebral osteomyelitis: a retrospective analysis using the Japanese diagnosis procedure combination database. BMJ Open.

[22] Berbari EF, Kanj SS, Kowalski TJ (2015). 2015 Infectious Diseases Society of America (IDSA) Clinical Practice Guidelines for the diagnosis and treatment of native vertebral osteomyelitis in adults. Clin Infect Dis.

